# Editorial: Tissue Remodeling in Health and Disease Caused by Bacteria, Parasites, Fungi, and Viruses

**DOI:** 10.3389/fcimb.2021.642311

**Published:** 2021-02-02

**Authors:** Sigrun Lange, Marcel I. Ramirez

**Affiliations:** ^1^ Tissue Architecture and Regeneration Research Group, School of Life Sciences, University of Westminster, London, United Kingdom; ^2^ Instituto Oswaldo Cruz, Rio de Janeiro, Brazil; ^3^ Departamento de Bioquímica e Biologia Molecular, Universidade Federal do Paraná, Curitiba, Brazil

**Keywords:** tissue remodeling, host manipulation, parasites, bacteria, fungi, viruses, zoonosis, host-pathogen interaction

## Introduction

Tissues undergo constant remodeling to maintain architecture during growth, in normal physiology and in response to disease. Interactions of the host with both commensals and pathogens, which include bacteria, viruses, fungi, and parasites, may affect not only immune responses due to recognition of pathogen associated molecular patterns (PAMPS), but also tissue remodeling including for example through the generation of neo-epitopes and damage-associated molecular patterns (DAMPS). Roles for the microbiome, viriome, fungi, as well as pathogenic bacteria and parasites, in both homeostasis and in host-pathogen interactions, is a topic of considerable interest regarding effects relating to chronic disease, cancer, dysbiosis, the gut-brain axis, host metabolism, drug metabolism, and zoonotic disease.

## Aims and Objectives

This Research Topic sought to collect state-of-the-art primary research studies and review articles from international experts and diverse groups in the field to further current understanding of the contributions of both commensals and pathogens in tissue remodeling in physiological and pathophysiological processes of the host.

## Findings

Ma et al. used serum lipidomics analysis of classical swine fever virus infection in piglets to investigate the emerging role of free fatty acids in virus replication. This shows impact of lipids metabolism in CSFV infection using *in vivo* and *in vitro* approaches, identifying 167 differentially expressed lipid metabolites in piglets infected with CSFV, also relating to metabolites effects on mitochondrial and IFN signaling pathways. These findings provide new understanding of specific lipid metabolites in virus replication.

Balasubramanian et al. identified that the association of *Plasmodium berghei* with the apical domain of hepatocytes is necessary for the parasite’s liver stage development. *Plasmodium* amplification in infected hepatocytes requires large-scale interactions with, and manipulations of, host cell functions. Using 3-D imaging and advanced image analysis of Plasmodium-infected liver tissues, the authors showed how the parasite is actively involved in modulating the architecture of the bile canicular liver tissue by associating with the apical domain of hepatocytes.

Sun et al. discuss differences in bacterial flora in pancreatic cancer versus normal pancreatic tissue and highlight putative roles for oral bacteria associated with pancreatic cancer and their virulence factors linked with cancer. This emphasizes novel preventive and therapeutic strategies based on bacterial and virulence factors in pancreatic cancer.

Liu et al. investigated the roles for Kupffer Cells as important participant of hepatic alveolar echinococcosis (AE), which is caused by *Echinococcus multilocularis* and is characterized by a large multilocular cyst with a jelly-like substance. This causes hepatomegaly and recurrent jaundice in patients. The authors showed that KC have immune-protective effect of anti-echinococcosis and promote liver fiber repair, indicating novel potential therapeutic value for patients with hepatic AE.

Daumas et al. assessed defective granuloma formation in elderly patients with sepsis by monitoring granuloma formation over the time course of infection with TB or *Coxiella burnetii*, compared with non-infected controls. The authors showed that impairment of granuloma formation was associated with reduced production of tumor necrosis factor without overproduction of interleukin-10, while furthermore genes specifically modulated in granulomatous cells were down-modulated in patients with defective granuloma formation.

Liao et al. investigate regenerative approaches in periodontitis, which is one of the most prevalent oral diseases, caused by bacterial infection and leads to destruction of alveolar bone. The authors generated scaffolds from mesoporous hydroxyapatite/chitosan, loaded with recombinant-human amelogenin, which exhibited antibacterial effects against both *Fusobacterium nucleatum* and *Porphyromonas gingivalis*, also promoting the formation of bone and cementum-like tissue.

Macedo-da-Silva et al. used serum proteomics approaches to investigate putative disease predictive biomarkers in infants with in utero exposure to Zika virus, but who were born without congenital Zika syndrome. Zika is one of the major zoonotic viruses and causes a range of, including severe, developmental neurological problems. The authors identified alterations in protease activity, axon guidance, and visual phototransduction proteome pathways in these infants, highlighting early biomarkers that are predictive for future late complications.

## Conclusion

The collection of articles presented here in this Research Topic is a small representation of research into host-pathogen/commensal interactions, highlighting the complexity of the interplay with the host in both health and disease. The co-evolution of pathogens and hosts must be considered, both in shaping the immune system throughout evolution, gut-brain axis communication, metabolism, chronic disease, and cancers, as well as in relation to zoonotic and emerging diseases.

## Author Contributions

SL: Writing original manuscript. MR: Manuscript writing—Review and editing. All authors contributed to the article and approved the submitted version.

## Conflict of Interest

The authors declare that the research was conducted in the absence of any commercial or financial relationships that could be construed as a potential conflict of interest.

